# Association between activity limitations and pain in patients scheduled for total knee arthroplasty

**DOI:** 10.1186/s12891-016-1233-2

**Published:** 2016-09-01

**Authors:** Ilana M. Usiskin, Heidi Y. Yang, Bhushan R. Deshpande, Jamie E. Collins, Griffin L. Michl, Savannah R. Smith, Kristina M. Klara, Faith Selzer, Jeffrey N. Katz, Elena Losina

**Affiliations:** Orthopaedic and Arthritis Center for Outcomes Research, Department of Orthopaedic Surgery, Brigham and Women’s Hospital, 75 Francis Street, BC-4016, Boston, MA 02115 USA

**Keywords:** Total knee arthroplasty, Osteoarthritis, Pain, Functional limitations

## Abstract

**Background:**

Historically, persons scheduled for total knee arthroplasty (TKA) have reported severe pain with low demand activities such as walking, but recent data suggests that TKA recipients may have less preoperative pain. Little is known about people who elect TKA with low levels of preoperative pain. To better understand current TKA utilization, we evaluated the association between preoperative pain and difficulty performing high demand activities, such as kneeling and squatting, among TKA recipients.

**Methods:**

We used baseline data from a randomized control trial designed to improve physical activity following TKA. Prior to TKA, participants were categorized according to Western Ontario and McMaster Universities Osteoarthritis Index (WOMAC) Pain scores: Low (0–25), Medium (26–40), and High (41–100). Within each group, limitations in both low demand and high demand activities were assessed.

**Results:**

The sample consisted of 202 persons with a mean age of 65 (SD 8) years; 21 %, 34 %, and 45 % were categorized in the Low, Medium, and High Pain groups, respectively. Of the Low Pain group, 60 % reported at least one of the following functional limitations: limited flexion, limp, limited walking distance, and limitations in work or housework. While only 12 % of the Low Pain group reported at least moderate pain with walking on a flat surface, nearly all endorsed at least moderate difficulty with squatting and kneeling.

**Conclusions:**

A substantial number of persons scheduled for TKA report Low WOMAC Pain (≤25) prior to surgery. Persons with Low WOMAC Pain scheduled for TKA frequently report substantial difficulty with high demand activities such as kneeling and squatting. Studies of TKA appropriateness and effectiveness for patients with low WOMAC Pain should include measures of these activities.

**Trial registration:**

Identifier NCT01970631; Registered 23 October 2013.

## Background

Total knee arthroplasty (TKA) is a common and effective surgery for end-stage knee osteoarthritis (OA), with over 600,000 TKAs performed each year in the US [1, 2]. Historically, only persons with severe pain, extensive structural changes, and limited range of motion have been considered candidates for TKA [3–5]. However, more recently, younger individuals reporting less severe pain on low demand activities such as walking are opting for TKA, and the number of younger persons electing to undergo the procedure is predicted to increase [6–10]. These trends raise questions about the characteristics of persons with low pain on basic activities who undergo TKA.

The Western Ontario and McMaster Universities Osteoarthritis Index (WOMAC) has been widely used to evaluate candidates for TKA and to measure the effectiveness of the surgery [11–14]. The WOMAC Pain scale asks about pain while lying down, sitting, standing, walking on a flat surface, and climbing stairs. While the activities captured by the WOMAC Pain scale range in difficulty, we refer to these items as “low demand” activities compared to more difficult mobility-related activities such as running and jumping, which we refer to as “high demand” activities. We also consider squatting, kneeling, and twisting to be high demand activities, as these methods of changing body position can be challenging for persons with knee conditions. These five high demand activities are measured by the Knee injury and Osteoarthritis Outcomes Score (KOOS) Sports and Recreational Activity scale.

Numerous studies have pointed to high WOMAC Pain prior to TKA as a risk factor for a poor outcome, as well as for lower satisfaction with the surgery [15, 16]. However, persons with low WOMAC Pain prior to surgery remain an understudied group in terms of their outcomes and expectations for surgery. Preoperative expectations have been shown to be associated with surgical satisfaction, although there are some conflicting reports about this connection, and no literature yet exists on how these expectations differ in patients with low WOMAC Pain prior to surgery [17–20]. In the absence of substantial pain with low demand activities, it is important to understand the characteristics of patients with low WOMAC Pain and what may be driving them to seek TKA.

Persons scheduled for TKA with low WOMAC Pain may have activity limitations in domains beyond those measured by the WOMAC Pain scale. While patients may not typically choose to undergo TKA in order to return to rigorous activities such as skiing or running, TKA recipients often report that movements such as kneeling and pivoting are important to their quality of life, and they may seek TKA in order to participate more fully or re-engage in recreational activities such as gardening [21–23].

Moreover, existing suggestions for TKA appropriateness criteria are limited and heavily weigh symptoms related to the execution of low demand activities [3, 24–26]. These reports consider patients without severe pain with walking or other low demand activities to be inappropriate candidates for TKA, regardless of age or radiographic severity [3, 25]. It is therefore unclear how to determine the appropriateness of TKA in persons with low WOMAC Pain, and such determinations require a better understanding of these patients’ characteristics prior to surgery. We hypothesized that TKA recipients reporting low levels of pain with low demand activities will be limited in high demand activities such as squatting and kneeling.

## Methods

### Study design

We analyzed preoperative baseline data from the Study of Physical Activity Rewards after Knee Surgery (SPARKS), a randomized controlled clinical trial (RCT) aimed at establishing the efficacy of a behavioral economics-based intervention for improving physical activity following TKA. The sample size for this proof of concept RCT was based on increases in physical activity post-TKA due to a behavioral intervention and was estimated at 200. Patients with knee OA scheduled to undergo a unilateral TKA at Brigham and Women’s Hospital (BWH) in Boston were enrolled from January 2014 to January 2016. Participants completed baseline questionnaires and wore Fitbit Zip accelerometers (Fitbit Inc, San Francisco, CA) for one week prior to TKA. The trial was approved by the Partners Healthcare Institutional Review Board and is registered on https://ClinicalTrials.gov (identifier NCT01970631).

### Enrollment

Persons scheduled to undergo a primary, unilateral TKA at BWH were eligible for the study if they were over 40 years old, had an underlying diagnosis of OA, were not planning to undergo another surgery within six months, did not have inflammatory arthritis, dementia, epilepsy, Parkinson’s disease, or neuropathy, and did not live in a nursing home. Subjects needed to be willing and able to use a Fitbit Zip accelerometer and to complete questionnaires online. Eligible subjects who agreed to participate met with a research assistant for a baseline visit, during which written informed consent was obtained and the patient was provided a Fitbit and instructions for its use.

### Assessments and outcome measures

The baseline questionnaire, which participants completed within 8 weeks of surgery, included demographic and clinical characteristics, quality of life, pain and functional status, and limitations in demanding recreational activities. Demographic information included age, sex, body mass index (BMI), race, education level, and employment status. We relied on the expert opinion of our orthopedic colleagues to identify the functional limitations that patients often cite as key reasons to undergo TKA. These included: inability to fully bend or extend knee, limp, limited walking distance, and pain interference with work or housework. Mental health was evaluated with the Mental Health Inventory (MHI-5), a 5-item questionnaire measuring anxiety and depressive feelings scaled from 0 to 100, with lower scores indicative of worse mental health [27, 28]. Health-related quality of life was calculated using the EuroQol EQ-5D-3L instrument, which is a self-rating of general health across five domains: mobility, self-care, usual activities, pain/discomfort, and anxiety/depression. Responses to each of the five domains were converted to a summary score on a 0 to 1 scale, with 1 representing the best quality of life, using published crosswalk index values [29]. Range of motion was self-reported using the validated method of Gioe and colleagues, in which study participants were presented with pictures of knees positioned at varying levels of flexion and extension [30, 31].

Pain and functional status was measured using the WOMAC Pain and Function scales [11]. Limitations in demanding recreational activities were measured using the Sport and Recreational Activity subscale of the Knee injury and Osteoarthritis Outcome Score (KOOS), which measures the difficulty that respondents experience with certain high demand activities (twisting, squatting, kneeling, jumping, and running) [22, 32]. Study participants were asked to rate the difficulty they experienced performing each of these five activities on a 5-level Likert scale ranging from no difficulty to extreme difficulty. A composite KOOS Sport and Recreational Activity subscore was calculated for participants who answered at least 3 of the 5 items [22]. Responses to the WOMAC Pain and Function scales and the KOOS Sport and Recreational Activity scale were scaled to range from 0 to 100, with 100 corresponding to the worst health status.

At the baseline visit, participants were asked to wear a Fitbit Zip accelerometer for seven consecutive days. An average number of daily steps was calculated using only the days with at least 8 h of wear time.

### Analytic approach

Subjects were stratified by preoperative WOMAC Pain level: Low (0–25), Medium (26–40), and High (41–100). The WOMAC Pain group cutoffs were made based on distributional assumptions and to increase the transparency of interpretation of pain group status. The cutoffs also avoid overstating a ‘dose-response’ relationship. Defining pain groups based on WOMAC pain <25, 26–40, and >40 had meaningful clinical interpretation. Almost all the patients (41 out of 43) with WOMAC Pain <=25 endorsed mostly none, mild or moderate pain on each item with at most one item above moderate pain. Most of the patients (40 out of 68) with WOMAC pain 26–40 endorsed moderate to extreme pain on at least two items, with at most three items with moderate to extreme pain. Those in the High pain group generally had to endorse moderate, severe or extreme pain, with 57 out of the 91 patients in this group endorsing moderate to extreme on all items. We evaluated the association between preoperative WOMAC Pain group and demographic features, clinical characteristics, and daily step count. We also evaluated the responses to each of the five individual items on both the WOMAC Pain and the KOOS Sport and Recreational Activity subscales. Demographic and clinical features were summarized as means and standard deviations (SD) for continuous variables and as proportions for categorical variables.

In order to assess functional limitations not captured by the WOMAC Pain subscale, we evaluated four clinically-meaningful characteristics: range of motion, limp, walking distance, and limitations in work or housework. We dichotomized each of these four variables to identify patients with clinically-relevant functional limitations: flexion ≤100°, at least moderate limp, limited to walking fewer than five blocks, or at least moderate limitations in work or housework. At least moderate limp and at least moderate limitations in work or housework included responses of moderate, severe, or extreme on a five-item Likert scale. We calculated the number of patients in each WOMAC Pain group who had 0, 1, or 2 or more of these four functional limitations.

Tests for trend across pain groups were conducted for demographic and clinical characteristics using the Jonckheere–Terpstra test for continuous variables and the Cochran–Mantel–Haenszel test for categorical variables. *P*-values reported in this manuscript refer to overall linear trends across the three WOMAC Pain groups. Statistical significance was indicated at a two-sided *p*-value less than 0.05. Statistical analysis was performed using SAS v9.4 (Cary, NC, USA).

## Results

### Sample characteristics

Two hundred fifty-one patients agreed to participate in the SPARKS study. Our study sample comprises the 202 participants who completed the baseline questionnaire, wore the Fitbit for the appropriate number of days, underwent surgery, and were ultimately randomized. Participants were 57 % female, had a mean age of 65 years (SD 8), and had a mean BMI of 31 (SD 6) (Table 1). Patients who were eligible for the study but did not agree to participate or could not be contacted had a mean age of 68 years (SD 9) and were 64 % female.

**Table 1:** Demographic characteristics of the sample of subjects scheduled for TKA by WOMAC pain group

	WOMAC Pain Group				*p*-value (trend)
	Low	Medium	High	Overall	
	(0–25)	(26–40)	(41–100)		
	*n* = 43 (21 %)	*n* = 68 (34 %)	*n* = 91 (45 %)	*n* = 202	
Age: mean (SD)	68 (7)	66 (8)	64 (7)	65 (8)	0.001
Female: no. (%)	16 (37 %)	38 (56 %)	61 (67 %)	115 (57 %)	0.001
BMI: mean (SD)	29 (5)	31 (6)	32 (6)	31 (6)	0.04
Race					0.06
White	41 (95 %)	63 (93 %)	78 (86 %)	182 (90 %)	
Non-White	2 (5 %)	5 (7 %)	13 (14 %)	20 (10 %)	
Education: no. (%)					0.04
Graduated from college	34 (79 %)	48 (71 %)	56 (62 %)	138 (68 %)	
Did not graduate from college	9 (21 %)	20 (29 %)	35 (38 %)	64 (32 %)	
Employment Status: no. (%)					0.34
Employed full- or part-time	24 (57 %)	40 (60 %)	44 (50 %)	108 (55 %)	
Not working	18 (43 %)	27 (40 %)	44 (50 %)	89 (45 %)	

The pain groups were as follows: 21 % Low Pain, 34 % Medium Pain, and 45 % High Pain. The mean age of the study subjects was 68 years (SD 7), 66 years (SD 8), and 64 years (SD 7) (*p* = 0.001) in the Low, Medium, and High Pain groups, respectively (Table 1). The Low Pain group was 63 % male, and the Medium and High Pain groups were 44 % and 33 % male, respectively (*p* = 0.001). The Low Pain group had a mean BMI of 29, and the Medium and High Pain groups had mean BMIs of 31 and 32, respectively (*p* = 0.04). Most participants were White: 95 % of the Low Pain group, 93 % of the Medium Pain group, and 86 % of the High Pain group (*p* = 0.06). Employment status was not associated with baseline WOMAC Pain group, with 57 % of Low Pain, 60 % of Medium Pain, and 50 % of High Pain participants reporting full or part time employment (*p* = 0.34). Baseline WOMAC Pain was associated with education, with 79 % of subjects in the Low Pain group, 71 % of the Medium Pain group, and 62 % of the High Pain group reporting having earned a bachelor’s degree (*p* = 0.04).

### Clinical characteristics

The overall mean WOMAC Pain score was 41 (SD 19), and the overall mean WOMAC Function score was 41 (SD 18) (Table 2). The mean WOMAC Function score for the Low Pain group was 23 (SD 11), 35 (SD 10) for the Medium Pain group, and 54 (SD 14) for the High Pain group (*p* <0.001). Health-related quality of life, as measured by the EQ-5D-3L, was 0.81 (SD 0.08) for the Low Pain group, 0.77 (SD 0.08) for the Medium Pain group, and 0.64 (SD 0.18) for the High Pain group (*p* <0.001).

**Table 2:** Clinical characteristics of the sample of subjects scheduled for TKA by WOMAC pain group

	WOMAC Pain Group				*p*-value (trend)
	Low	Medium	High	Overall	
	(0–25)	(26–40)	(41–100)		
	*n* = 43 (21 %)	*n* = 68 (34 %)	*n* = 91 (45 %)	*n* = 202	
WOMAC Pain: mean (SD)	16 (7)	35 (4)	58 (14)	41 (12)	<0.001
WOMAC Function: mean (SD)	23 (11)	35 (10)	54 (14)	41 (18)	<0.001
HRQoL (EQ-5D-3 L Index): mean (SD)	0.81 (0.08)	0.77 (0.08)	0.64 (0.18)	0.72 (0.15)	<0.001
KOOS Sport and Activity: mean (SD)	63 (20)	68 (24)	84 (19)	74 (23)	<0.001
Knee extension: no. (%)					0.006
More than 5^0^ from straight	19 (44 %)	36 (54 %)	62 (68 %)	117 (58 %)	
Completely straight	24 (56 %)	31 (46 %)	29 (32 %)	84 (42 %)	
Knee flexion: no. (%)					0.046
100^0^ or less	5 (12 %)	11 (16 %)	23 (25 %)	39 (19 %)	
More than 100^0^	38 (88 %)	57 (84 %)	68 (75 %)	163 (81 %)	
Limp: no. (%)					0.001
Moderate to severe	15 (35 %)	29 (43 %)	57 (63 %)	101 (50 %)	
None to slight	28 (65 %)	39 (57 %)	34 (37 %)	101 (50 %)	
Walking distance: no. (%)					<0.001
Less than 5 blocks	8 (19 %)	26 (38 %)	54 (59 %)	88 (44 %)	
5 to 20 blocks	20 (48 %)	31 (46 %)	30 (33 %)	81 (40 %)	
Unlimited	14 (33 %)	11 (16 %)	7 (8 %)	32 (16 %)	
How much did pain interfere with work or housework?: no. (%)					<0.001
Moderately to extremely	15 (35 %)	43 (63 %)	79 (87 %)	137 (68 %)	
Not at all to a little bit	28 (65 %)	25 (37 %)	12 (13 %)	65 (32 %)	
Use of a supportive device: no. (%)					0.10
Yes	9 (21 %)	15 (22 %)	30 (33 %)	54 (27 %)	
No	34 (79 %)	53 (78 %)	61 (67 %)	148 (73 %)	

### Functional limitations

**Fig. 1:**
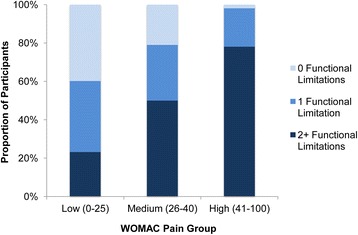
The proportion of participants in each WOMAC Pain group reporting 0, 1, and 2 or more functional limitations. The four basic functional limitations analyzed include poor range of motion (self-reported flexion greater than or equal to 100^0^), limitations in work or housework (moderate or greater limitations), limp (moderate or greater), or being unable to walk more than 5 blocks. The number of these functional limitations reported (0, 1, or 2 or more) was associated with WOMAC Pain group (*p* < 0.001)

We assessed four functional limitations: poor range of motion (flexion ≤100°), at least moderate limp, limited to walking less than five blocks, or at least moderate limitations in work or housework. Of those in the Low Pain group, 12 % reported flexion ≤100°, 35 % reported having at least a moderate limp, 19 % reported walking limited to five blocks, and 35 % reported at least moderate limitations in work or housework. Sixty-one percent of the Low Pain group experienced at least one functional limitation, and 23 % percent of participants in this group experienced at least two functional limitations. Of the Medium Pain group, 79 % experienced at least one and 50 % experienced at least two functional limitations. Ninety-eight percent of the High Pain group reported at least one functional limitation, with 78 % reporting two or more functional limitations (Fig. 1).

Thirty-five percent of the Low Pain group indicated that their pain at least moderately interfered with their regular work or housework, as did 63 % of the Medium Pain group and 87 % of the High Pain group (*p* <0.001). Pain was associated with knee extension, or the ability to completely straighten one’s knee (Low Pain: 56 %, Medium Pain: 46 %, High Pain: 32 %; *p* = 0.006). Worse pain corresponded to less knee flexion, with 88 % of the Low Pain group able to bend their knee more than 100°, while only 84 % of the Medium Pain group and 75 % of the High Pain group could bend their knee more than 100° (*p* = 0.046).

### Activity limitations

**Fig. 2:**
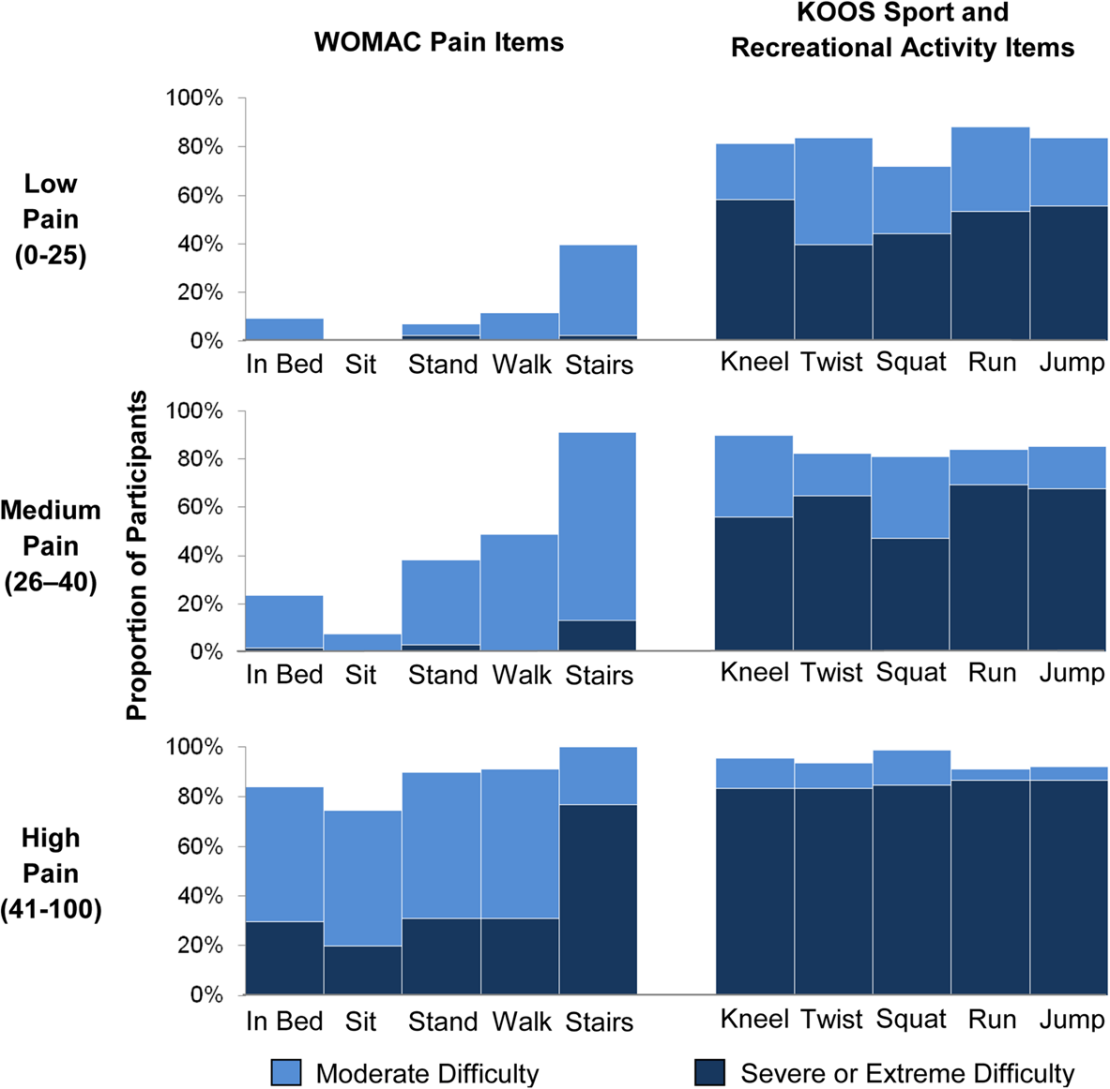
The proportion of participants scheduled to undergo TKA reporting difficulty performing basic tasks (measured by the WOMAC Pain scale) and more demanding activities (measured by the KOOS Sport and Recreational Activity subscale), stratified by level of WOMAC Pain. The dark blue portions of the bars represent patients who expressed severe or extreme difficulty performing each of the WOMAC Pain or KOOS Sport and Recreational Activity items. The light blue portions of the bars show patients who reported moderate difficulty with these same items. The total height of the bars represents the proportion of patients in each WOMAC Pain group who expressed moderate or greater difficulty performing each of the tasks

The mean KOOS Sport and Recreational Activity score was 63 (SD 20) for the Low Pain group, 68 (SD 24) for the Medium Pain group, and 84 (SD 19) for the High Pain group (*p* <0.001). A considerable proportion of the subjects in the Low Pain group experienced severe or extreme difficulty performing the high demand activities measured by the KOOS Sport and Recreational Activity scale: 58 % with kneeling, 40 % with twisting, 44 % with squatting, 54 % with running, and 56 % with jumping (Fig. 2). Two-thirds of the Low Pain group, 81 % of the Medium Pain group, and 98 % of the High Pain group reported severe difficulty with at least one of the five activities measured by the KOOS Sport and Recreational Activity scale.

When we broadened the definition of difficulty with tasks to include moderate (as well as severe and extreme) difficulty, the proportion of the Low Pain group experiencing difficulty with the KOOS Sport and Recreational Activity items increased to 81 % with kneeling, 84 % with twisting, 72 % with squatting, 88 % with running, and 84 % with jumping (Fig. 2). Every individual in the Low Pain group reported at least moderate difficulty performing at least one of these five high demand activities.

## Discussion

This paper reports the functional and activity limitations among persons scheduled for TKA, stratified by preoperative pain level. About one-fifth of subjects in the cohort reported Low WOMAC Pain scores (0–25) prior to surgery. However, these subjects frequently reported functional limitations such as limping or poor range of motion, as well as limitations with the high demand activities measured by the KOOS Sport and Recreational Activity scale, such as twisting, squatting, and kneeling.

Persons scheduled for TKA have been found to express goals of performing more than just low demand activities following surgery, such as returning to sports or gardening [23, 33]. A report by Noble and colleagues found that persons scheduled for TKA tend to place high importance on biomechanically-demanding activities such as kneeling and squatting, and that satisfaction with surgery is associated with their ability to return to the activities that they deem most important [18]. Our results corroborate these and other similar findings that TKA recipients may consider improvement in the ability to engage in more demanding activities such as kneeling or gardening as being important to their decisions to undergo TKA [22, 23, 33].

Despite evidence that many TKA recipients place high importance on demanding activities, data that support TKA recipients being able to engage in high demand activities such as kneeling, squatting, and twisting after surgery are limited [19, 22, 34]. Roos and colleagues evaluated the ability of subjects to perform the activities on the KOOS Sport and Recreational Activity subscale both pre-operatively and six months post-TKA and found that TKA provided only modest increases in the number of subjects who reported being able to squat, run, jump, and twist, and decreases in the number who reported being able to kneel [22]. Additionally, Weiss and colleagues found that patients often regard kneeling and gardening as some of the most important but also some of the most difficult activities to perform following TKA [23]. The increasing numbers of patients with low WOMAC Pain electing TKA highlights a need for more research that uses post-TKA data to evaluate the benefits of surgery specifically for patients with low WOMAC Pain. If patients are motivated to undergo TKA not by limitations in low demand activities but by the desire to return to more demanding activities, more attention should be paid to outcomes for patients with low preoperative pain that are related to performing these high demand activities. Moreover, persons with low WOMAC Pain who opt to undergo TKA may benefit from additional discussions with their surgeon regarding expectations of returning to such activities. Thorough discussions about managing expectations before indicating TKA may help to alleviate concerns about patients with low pain prior to surgery expecting improvements in high demand activities.

Additionally, future research on appropriateness criteria for TKA should account for patients who report low WOMAC Pain but who may seek surgery as a way to return to the activities that they deem important for their quality of life. Previous work on developing appropriateness criteria for TKA has included factors such as age, preoperative pain and function, and radiographic findings. Escobar and colleagues created criteria for TKA based on the RAND/UCLA Appropriateness Method, where a panel of experts rated cases as inappropriate, inconclusive, or appropriate [3]. The resulting criteria deemed patients with mild or moderate symptoms inappropriate or uncertain candidates for TKA regardless of age or radiographic severity, where moderate symptoms were defined as pain when walking on level surfaces and having some limitation in daily activities. Using Escobar’s criteria, Riddle and colleagues deemed over half of 175 TKA recipients in the Osteoarthritis Initiative (OAI) to be inappropriate or inconclusive TKA candidates [3, 5]. Hawker and colleagues used a cutoff of 39 points on the combined WOMAC Pain and Function scales (out of 100 points, 100 worst) to identify patients who had OA symptoms severe enough for TKA [25]. An evaluation conducted by Ghomrawi and colleagues found poor agreement between the criteria used by Escobar and Hawker, demonstrating a critical need for consistent and relevant appropriateness standards for TKA [24].

Fifty-five percent of SPARKS participants had WOMAC Pain below 40 points, and would likely not be considered appropriate TKA candidates based on several proposed appropriateness criteria [3, 25]. These data are consistent with the assessment by Riddle and colleagues that deemed only 44 % of 175 patients in the OAI to be appropriate TKA recipients based on Escobar’s criteria [3, 5]. The substantial number of TKAs in our patient sample and in the OAI that would likely be considered inappropriate based on Escobar’s criteria highlights the mismatch between these criteria developed almost fifteen years ago and current practice [3].

Additionally, the substantial number of patients with low WOMAC Pain scheduled for TKA suggests that the WOMAC Pain scale is an insufficient measure of TKA appropriateness, as has been previously described [35–37]. Researchers have attempted to use other measures such as the KOOS Pain and Function subscales to aid in the assessment of TKA appropriateness, but the KOOS Sport and Recreational Activity subscale has not been explored in this capacity [35]. The use of computerized adaptive testing may be a potential option for overcoming the limitations of the WOMAC for measuring a wide range of activity and function limitations for persons considering TKA. For example, PROMIS computerized adaptive testing has been used to measure self-reported physical function in patients with arthritis and in orthopedic trauma patients [38, 39].

In the development of appropriateness criteria for TKA, it is important to recognize that pain may not be the primary focus for patients. There may be other factors besides pain on the WOMAC Pain items, such as BMI, that contribute to limitations in the high demand activities measured by the KOOS Sport and Recreational Activity subscale. In the development of appropriateness criteria for TKA, it is important to recognize that pain may not be the primary focus for patients. More work is needed to develop appropriateness criteria that account for the interplay between pain and other variables such as demographic characteristics and activity limitations. The relevance of our hypothesis that TKA recipients who report low levels of pain with low demand activities will be limited in high demand activities lies in fact that WOMAC Pain relies largely (3 out 5 items) on sedentary activities and therefore could miss the disability of the increasingly active population of TKA candidates.

We found that participants with Low Pain prior to surgery did not differ from those with Medium or High Pain with regard to the average number of steps they walked every day (Table 2). This finding is similar to that of White and colleagues, who reported that knee pain severity did not impact walking behaviors in a cohort with or at risk for knee OA [40]. Lo and colleagues also recently showed that WOMAC Pain scores did not predict physical activity levels among OAI participants with or without knee OA [41]. It is somewhat paradoxical that participants who report low WOMAC Pain and therefore experience less pain when standing or walking on flat surfaces do not walk more than those with more pain. This conveys discordance between potential capacity and performance that is often observed in knee OA cohorts, where participants who can walk without pain nonetheless choose not to [40, 41]. It is also possible that participants with low WOMAC Pain do not walk more than those with more pain because they have modified their activity to be in less pain. Our findings were not affected by missing data, since completing the baseline assessment was a key inclusion criterion for the study.

The results of this study should be viewed within the context of several limitations. The study population was recruited as a part of randomized controlled clinical trial, which introduces inherent selection bias, and the participants were recruited from a single study center. Additionally, because the study sample was obtained from a randomized controlled trial of a behavioral intervention for physical activity following TKA, subjects with low pain may have been more willing to participate. This selection bias may have enriched the proportion of subjects in our sample with low WOMAC Pain, allowing us to examine their characteristics more carefully. Individuals with severe mobility limitations were excluded from the study, and thus our sample may be more active than other TKA cohorts. This study did not include radiographs, and thus we were unable to determine the radiographic severity of subjects’ knee OA, which could have influenced decisions to pursue TKA. Knee range of motion was obtained using self-report; however, participant-reported knee range of motion has been shown to match measured range of motion in a similar population with knee OA [31]. This analysis also uses single items from multi-item scales (the KOOS and the WOMAC), which have unknown validity and may compromise the reliability of the results. Our questionnaire did not give participants the option to indicate that they did not perform the high demand activities measured by the KOOS, which may have led some participants to report “extreme” difficulty with activities that they do not perform. Functional limitation items were selected based on expert opinion and were not extensively validated. In addition, we did not collect data related to motivation for TKA. Future studies should directly measure patient motivations for undergoing TKA and how satisfied they are with surgery in order to better understand why patients with low pain on low demand activities undergo TKA.

## Conclusions

About one out of five subjects from the SPARKS study sample had WOMAC Pain ≤25 prior to TKA. Those with Low Pain frequently reported severe or extreme difficulty performing high demand activities, such as kneeling or squatting. We suggest that future work on determining appropriateness criteria for TKA should consider limitations beyond the low demand activities measured by the WOMAC Pain scale. Additionally, it is important that patients and surgeons discuss preoperative expectations to ensure that patients have reasonable expectations for returning to demanding activities following surgery. More research is needed to understand what motivates patients with low WOMAC Pain to seek TKA and how to measure surgical effectiveness in such patients.

## Abbreviations

BMI: Body Mass Index
BWH: Brigham and Women’s Hospital
KOOS: Knee injury and Osteoarthritis Outcome Score
MHI-5: Mental Health Inventory
OA: Osteoarthritis
OAI: Osteoarthritis Initiative
SD: Standard deviation
SPARKS: Study of Physical Activity Rewards After Knee Surgery
TKA: Total knee arthroplasty
WOMAC: Western Ontario and McMaster Universities Osteoarthritis Index
